# An Update on Intraocular Lens Technology for Presbyopia Correction and Visual Outcomes

**DOI:** 10.3390/medsci14020299

**Published:** 2026-06-10

**Authors:** Ava Niknahad, Grzegorz Łabuz, Maria Muzyka-Woźniak, Timur M. Yildirim, Hyeck-Soo Son, Gerd U. Auffarth

**Affiliations:** 1 The David J. Apple Center for Vision Research, Department of Ophthalmology, University of Heidelberg, 69120 Heidelberg, Germany; aniknah1@jh.edu (A.N.); grzegorz.labuz@med.uni-heidelberg.de (G.Ł.); timur.yildirim@med.uni-heidelberg.de (T.M.Y.); hyecksoo.son@med.uni-heidelberg.de (H.-S.S.); 2 Research and Development Center CREO, Ophthalmology Clinical Center SPEKTRUM, 53-334 Wroclaw, Poland; mmw@spektrum.wroc.pl

**Keywords:** intraocular lens, presbyopia, monofocal IOL, monofocal plus IOL, extended depth of focus IOL, trifocal IOL

## Abstract

Background/Objectives: Choosing the most appropriate intraocular lens (IOL) for presbyopia management may be challenging given the expanding selection of available designs. This review provides an updated overview of current monofocal plus, extended depth of focus (EDoF), and trifocal IOLs, summarizing their optical properties and laboratory and clinical findings, including visual acuity outcomes and side effects. Methods: A literature search was conducted using PubMed to identify studies on monofocal plus, EDoF, and trifocal IOLs, with emphasis on optical characteristics, visual acuity outcomes, and reported photic phenomena. Results: Monofocal plus IOLs demonstrate an improvement in depth of field compared to monofocal lenses, as evidenced by significant broadening of the defocus curve. Both refractive and diffractive EDoF IOLs show improved intermediate visual acuity, with diffractive models offering greater depth of field but a higher risk of dysphotopsia. Trifocal IOLs offer the best visual acuity at all three foci: near, intermediate, and distance. Patients’ needs may be customized using sulcus- or capsulotomy-fixated IOLs, mix-and-match strategies, and binocular IOL systems. Conclusions: Based on this literature review, clinical and optical bench studies to date support optimized IOL selection based on individual patient needs for presbyopia management. However, consideration of understudied or newly released IOL models is limited as future research is needed. Additionally, further prospective, randomized, controlled, and masked studies with large sample sizes on all IOL models may further support patient decision-making by contributing to a more comprehensive literature.

## 1. Introduction

**Figure 1:**
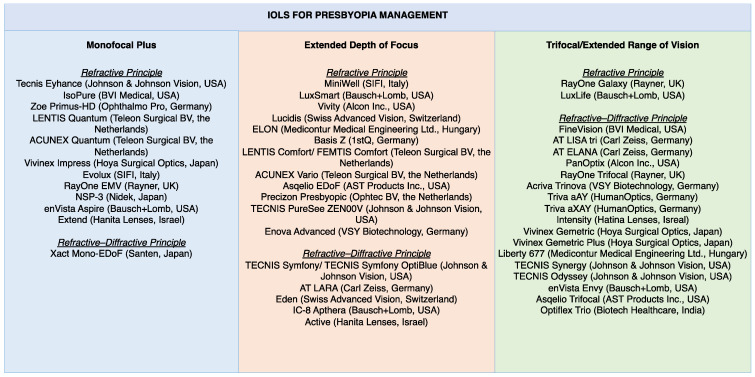
List of the discussed intraocular lenses (IOLs) for presbyopia management categorized by IOL design.

Since their introduction in 1949, intraocular lenses (IOLs) have enabled vision restoration after lensectomy [1]. While once designed as monofocal lenses that provide good visual acuity at a distance, the technology of their optics has evolved to provide patients with good visual acuity over a range of distances. The development of such newer lenses, typically referred to as presbyopia-correcting IOLs, such as extended depth of focus (EDoF) and trifocal lenses, is especially helpful in compensating for the lack of natural accommodation after lensectomy. To help physicians and patients choose an IOL from the multitude of manufacturers and their designs, this review aims to describe studies that evaluate lenses alone or against one another and contextualize their outcomes in real-world scenarios using clinical data. By doing so, we hope to provide the most up-to-date information on the various presbyopia-correcting IOLs, with a review of their available literature up to mid-May 2025. A list of the monofocal plus, extended depth of focus, and trifocal/extended range of vision IOLs discussed in the review is provided in Figure 1. Characteristics and visual acuity findings of the IOLs are respectively listed in Table 1 and Table 2 for monofocal plus IOLs, in Table 3 and Table 4 for EDoF IOLs, and in Table 5 and Table 6 for trifocal IOLs. It should be noted that our classification of IOLs for presbyopia management does not currently follow the recently introduced 2024 European Society of Cataract and Refractive Surgeons (ESCRS) functional classifications, as these are not yet widely adopted in clinical practice, and applying this new classification to the presented models requires further research [2].

## 2. Methods

To identify the IOLs for inclusion in this review, a literature search was conducted using PubMed with each of the following search terms, either as intraocular lens, IOL, or lens: monofocal plus, extended depth of focus, EDoF trifocal, and extended range of vision. Mentions in the article’s title or abstract were searched. The search was conducted to include any published studies until 15 May 2025. Inclusion criteria allowed studies on any of the above IOLs that included clinical or optical bench testing information. Clinical case reports regarding patients who had the above IOL implants with the case irrelevant to their visual outcome or side effects were excluded. After all lenses were identified via PubMed, each lens was searched on its manufacturer’s website to include information on its properties and/or trials.

## 3. Monofocal Plus Lenses

After the introduction of the first IOLs, standard spherical monofocal lenses were used until they evolved into a more complex optical design featuring aspheric surfaces and “monofocal plus” technology to enhance their outcomes. Aspheric surfaces mitigate the effects of corneal spherical aberration (SA) and have shown a significant influence on IOL performance [3]. Aspheric IOLs are designed with specific spherical aberrations to either preserve or compensate for the patient’s native ocular SA, offering SA compensation up to −0.27 μm. Given that personalized SA correction is not yet widely available, various aspheric options allow choosing an IOL that is tailored to the patient’s corneal properties and can improve postoperative outcomes. For example, in patients after surgical myopic laser correction, the increase in SA values may be better compensated with aspheric IOLs with a higher SA correction, and vice versa being true for patients after surgical hyperopic correction [4]. On the other hand, monofocal plus or enhanced monofocal lenses were created to establish good distance visual acuity and improve intermediate visual acuity compared to standard monofocal IOLs, while minimizing photic phenomena typical of trifocal IOLs. Although a purely refractive design is the most common, a diffractive structure with low add power and a limited number of diffractive rings can also be used. The most popular approach, however, utilizes localized changes in IOL asphericity, which effectively provide additional power that often changes progressively as the pupil size enlarges. This modified area is often confined to the central 1–2 mm of the optic, in order to minimize any potential impact on distance vision, to keep performance close to that of a standard monofocal lens, and to ensure a close profile with respect to IOL-related photic phenomena. A summary of the characteristics of the monofocal plus IOLs, such as their material, optical design, optics diameter and spherical aberrations, is included in Table 1. A summary of the select studies on these monofocal plus lenses, when available, is included in Table 2.

**Table 1:** Overview of monofocal plus intraocular lenses’ characteristics.

Model	Manufacturer	Material	Optics Diameter/ Overall Diameter (mm)	Optical Design	Spherical Aberrations	Available Strengths (Steps) (Diopter)	Availability of Toric Versions
Tecnis Eyhance	Johnson & Johnson Vision	Hydrophobic acrylate	6.0/13.0	Refractive	−0.27 μm	+5.0 to +34.0 (0.5)	Yes (Tecnis Eyhance Toric II)
IsoPure	BVI Medical	Hydrophobic acrylate	6.0/11.0 5.75/10.75	Refractive	−0.11 μm	+10.0 to +24.5 (0.5) +25.0 to +30.0 (0.5)	Yes (IsoPure Serenity Toric)
Zoe Primus-HD	Ophthalmo Pro	Hydrophobic acrylate	6.0/13.0	Refractive	−0.20 μm	−10.0 to +36.0 (0.5)	Yes (Primus-HD Toric)
LENTIS Quantum	Teleon Surgical BV	Hydrophilic acrylate	6.0/11.0	Refractive	Aberration-neutral	+10.0 to +30.0 (0.5)	No
ACUNEX Quantum	Teleon Surgical BV	Hydrophobic acrylate	6.0/12.5	Refractive	Aberration-neutral	+10.0 to +30.0 (0.5)	No
Vivinex Impress	Hoya Surgical Optics	Hydrophobic acrylate	6.0/13.0	Refractive	−0.18 μm	+6.0 to +30.0 (0.5)	Yes (Vivinex Toric)
Evolux	SIFI	Hydrophobic acrylate	6.0/13.0	Refractive	Aberration-neutral	+5.0 to +10.0 (1.0) +10.5 to +30.0 (0.5)	No
RayOne EMV	Rayner	Hydrophilic acrylate	6.0/12.5	Refractive	Aberration-neutral	+10.0 to +30.0 (0.5)	Yes (RayOne EMV Toric)
NSP-3	Nidek	Hydrophilic acrylate	6.0/13.0	Refractive	Not available	+1.0 to +5.0 (1.0) +5.0 to +27.0 (0.5) +27.0 to +30.0 (1.0)	No
enVista Aspire	Bausch + Lomb	Hydrophilic acrylate	6.0/12.5	Refractive	Aberration-neutral	+6.0 to +34.0 (0.5)	Yes (enVista Aspire Toric)
Extend SL	Hanita	Hydrophilic acrylate	6.0/13.0	Refractive	−0.13 μm	+5.0 to +30.0 (0.5) +30.0 to +34.0 (1.0)	Yes (Extend SL Toric)
Xact Mono-EDoF	Santen	Hydrophilic acrylate	6.0/12.5	Refractive–diffractive	−0.17 μm	+10.0 to +30.0 (0.5)	No

**Table 2:** Summary of select studies on monofocal plus intraocular lenses regarding their visual acuity findings.

Publication (Author, Year)	IOL Model	No. of Eyes	NVA (logMAR)	IVA (logMAR)	DVA (logMAR)
Baur, 2021 [5]	Xact Mono-EDoF	47	-	0.31 ± 0.12	−0.07 ± 0.08
Giglio, 2024 [6]	TECNIS Eyhance	60	0.25 ± 0.13	0.17 ± 0.13	−0.03 ± 0.05
Mencucci, 2023 [7]	IsoPure 123	24	0.408 ± 0.090	0.210 ± 0.085	0.02 ± 0.04
Akahoshi, 2025 [8]	IsoPure Serenity	38	0.35 ± 0.14	0.28 ± 0.13	−0.08 ± 0.05
Mencucci, 2023 [7]	Eyhance	24	0.457 ± 0.115	0.231 ± 0.074	0.03 ± 0.04
Mencucci, 2023 [7]	Vivinex Impress	24	0.433 ± 0.067	0.222 ± 0.035	0.03 ± 0.04
Cano-Ortiz, 2024 [9]	Evolux	32	0.36 ± 0.16	0.18 ± 0.12	0.01 ± 0.06
García-Bella, 2024 [10]	RayOne EMV	50	-	0.28 ± 0.07	−0.03 ± 0.06
Feltrin de Barros, 2025 [11]	enVista Aspire	29	0.28	0.23	0.00

All studies are cited in the References. The visual acuities are all distance-corrected, monocular measurements. IOL: intraocular lens; No.: number; NVA: near visual acuity; IVA: intermediate visual acuity; DVA: distance visual acuity.

The first-introduced monofocal plus model was the Xact Mono-EDoF (Santen, Osaka, Japan) [5]. The Xact IOL is the only refractive–diffractive monofocal plus IOL, with four rings within the lens center. It has shown a corrected distance visual acuity (CDVA) of −0.09 ± 0.09 logMAR, and distance-corrected intermediate visual acuity (DCIVA) at 70, 60, and 50 cm of 0.25 ± 0.14, 0.31 ± 0.12, and 0.38 ± 0.15 logMAR, respectively [5]. All other lenses include a central lens area that provides an increased central refractive power. TECNIS Eyhance (Johnson & Johnson Vision, Irvine, CA, USA)—a refractive IOL—was one of the first such lenses that popularized the term monofocal plus [12]. Building on the TECNIS ZCB00 platform, the Eyhance includes a higher-order aspherical component in its central 2 mm region that leads to an increased refractive power of approximately 0.5 D [13,14], with many studies extensively evaluating it to date [15]. In optical bench studies, the difference between the standard and such monofocal plus IOLs is evident [16]. In an optical bench study comparing the standard monofocal lens TECNIS ZCB00 (Johnson & Johnson Vision, Irvine, CA, USA) to various monofocal plus models, TECNIS ZCB00 showed a peak corresponding to the distance focus with a higher modulation transfer function (MTF) compared to the TECNIS ICB00 (Eyhance, Johnson & Johnson Vision, Irvine, CA, USA), although the Eyhance model showed a secondary peak that merged with the primary focus due to its proximity [16]. Clinically, both randomized studies and case series have shown a DCIVA ranging from 0.07 to 0.28 logMAR, with an increase of 0.11 logMAR in visual acuity in the defocus curve compared to the TECNIS ZCB00 [6,12,17,18,19]. Studies using Eyhance have additionally shown that such IOL efficiency may decrease with increased pupil size, considering that only a limited area of the lens optics is optimized to improve depth of field in the intermediate vision [20].

After TECNIS Eyhance, many other refractive IOLs were introduced that took advantage of similar refractive designs. The IsoPure (BVI Medical, Waltham, MA, USA) is one such lens that affects the anterior and posterior surfaces with changes similar to aspheric properties up to the 10th order [21]. Studies on IsoPure so far have shown uncorrected binocular intermediate vision of at least 20/25 in 81% and 50% of patients at 80 and 66 cm, respectively [22]. Another IsoPure model, IsoPure Serenity, combines the IsoPure 123 model with a double C-loop posterior angulated haptic platform. In a prospective study by Akahoshi, patients implanted bilaterally with IsoPure Serenity were reported to have a mean monocular CDVA, DCIVA at 80 cm, DCIVA at 66 cm, and distance-corrected near visual acuity (DCNVA) of −0.08 ± 0.05, 0.23 ± 0.13, 0.28 ± 0.13, and 0.35 ± 0.14 logMAR, respectively [8].

The Zoe Primus-HD (Ophthalmo Pro, St. Ingbert, Germany) contains a central excess of SA that gradually decreases towards the periphery to create a greater depth of field [16]. LENTIS and ACUNEX Quantum (Teleon Surgical BV, Spankeren, The Netherlands) are two IOLs that utilize local addition with an additional power of +2.5 D that gradually decreases to reach a specific power within 1.5 mm. The lenses differ in their haptic configuration and base polymer [23]. The Vivinex Impress (Hoya Surgical Optics, Tokyo, Japan) modulates the power over a 2 mm intermediate region. In a retrospective study, Mencucci et al. demonstrated no significant differences in the visual outcomes of patients after implantation of Eyhance, Vivinex Impress, and IsoPure, as the monocular DCIVA was 0.23 ± 0.07, 0.22 ± 0.04, and 0.21 ± 0.09 logMAR, respectively [7].

The Evolux (SIFI, Catania, Italy) modulates the IOL power over a 4.5 mm region using the concept of opposite-sign SAs. In a clinical study of Evolux in 18 patients, patients demonstrated good uncorrected visual acuity outcomes with mean logMAR values of −0.04 for distance, 0.15 for intermediate, and 0.35 for near vision [9]. In optical bench studies, the Evolux IOL showed the broadest depth of focus compared to TECNIS Eyhance IOL and standard monofocal IOL AcrySof IQ [24]. The RayOne EMV (Rayner, Worthing, West Sussex, UK) produces an excess positive SA, which may be useful for patients who have previously received laser surgery for hyperopic treatment [25]. In vitro experiments have shown RayOne EMV to have better contrast and depth of focus in high-power lenses compared to spherical lenses [25]. In a prospective clinical study, RayOne EMV showed a logMAR visual acuity of 0.28 ± 0.07 in the intermediate range [10]. Another lens, NSP-3 (NIDEK, Gamagori, Japan), has shown a refractive tolerance on the myopic side up to 1.12 D [26]. The enVista Aspire IOL (Bausch + Lomb, Bridgewater, NJ, USA) expands the depth of focus through a 1.5 mm zone of variable curvature placed on the posterior surface. In a recent prospective study by Feltrin de Barros et al., monocular visual outcomes showed that at distance, 100% of eyes achieved 20/30 or better, and 93.1% achieved 20/25 or better. At intermediate distance, 10.3% achieved 20/20 or better, 62.1% achieved 20/30 or better, and 89.7% achieved 20/40 or better. At the near focus, 51.7% achieved 20/30 or better, and 82.8% achieved 20/40 or better, with the remaining eyes performing below these levels [11]. The Extend IOL (Hanita, Israel) utilizes a conical Axicon surface to improve intermediate vision. However, as of the current literature search, no peer-reviewed clinical or laboratory studies have been published on its effectiveness.

Lastly, regarding the side effects, the enhanced monofocal IOLs still show dysphotopsia similar to the other monofocal IOLs. Looking at the point spread function, which is the spread of light around a light source, the monofocal plus IOLs had minimally increased spread; e.g., Eyhance showed only an 8% increase in point spread function compared to a monofocal control [16]. Such a minute difference cannot be detected in clinical evaluation, as a similar occurrence of photic phenomena among standard and monofocal plus patients has been reported [12,18,19].

## 4. EDoF Lenses

EDoF lenses have been defined by the American Academy of Ophthalmology to be IOLs that offer a mean monocular photopic best-corrected distance VA that is non-inferior to a monofocal lens, and an improvement in depth of field of at least 0.5 D compared to a monofocal lens at 0.2 logMAR or a DCIVA of 0.2 logMAR or better at 66 cm in at least half of the examined eyes with statistically significant improvement over a monofocal IOL [27]. EDoF IOL design can be categorized into two main groups of refractive and diffractive. As found by optical bench studies looking at EDoF IOLs’ optical quality using MTF analysis at 50 lp/mm, refractive models exhibit a balanced energy distribution between distance and intermediate vision with a gradual decrease in their optical quality with increased defocus [28]. However, diffractive lenses produce two distinct focal points [28]. In optical bench studies, diffractive EDoF models have been found to increase the light intensity by point spread function, suggesting that they are associated with a higher probability of perceiving unwanted photic phenomena compared to refractive EDoF lenses [28]. Compared to diffractive trifocals, in a prospective study, Monaco et al. reported similar risk of dysphotopsia between a diffractive EDoF (Symfony) and a diffractive trifocal (Panoptix) [29]. Both diffractive and refractive EDoF models have been found to have a higher risk of this side effect compared to monofocal and monofocal plus IOLs [13,14]. A summary of the characteristics of the EDoF IOLs is included in Table 3. A summary of the selected studies on these lenses, when available, is included in Table 4.

**Table 3:** Overview of extended depth of focus intraocular lenses’ characteristics.

Model	Manufacturer	Material	Optics Diameter/ Overall Diameter (mm)	Optical Design	Spherical Aberrations	Available Strengths (Steps) (D)	Availability of Toric Versions
MiniWell	SIFI	Hydrophilic acrylate	6.0/10.75	Refractive	Aberration-neutral	0 to +10.0 (1.0) +10.5 to +30.0 (0.5)	Yes (MiniWell Toric)
LuxSmart	Bausch + Lomb	Hydrophobic acrylate	6.0/11.0	Refractive	Aberration-neutral	0 to +10.0 (1.0) +10.0 to +34.0 (0.5)	Yes (LuxSmart toric)
Vivity (Clareon)	Alcon Inc.	Hydrophobic acrylate	6.0/13.0	Refractive	−0.20 μm	+10.0 to +30.0 (0.5)	Yes (Vivity Toric)
Lucidis	Swiss Advanced Vision	Hydrophilic acrylate	6.0/10.8 6.0/12.4	Refractive	Not available	+5.0 to +30.0 (0.5)	Yes (Lucidis MT)
ELON	Medicontur	Hydrophobic acrylate	6.0/13.0	Refractive	Aberration-neutral	+10.0 to +30.0 (0.5)	Yes (ELON Toric)
Basis Z	1stQ	Hydrophilic acrylate	6.0/13.0	Refractive	Aberration-neutral	−10.0 to +45.0 (in 1.0) except +10.0 to +30.0 (0.5)	Yes (Basis Z Toric)
LENTIS Comfort/ FEMTIS Comfort	Teleon Surgical BV	Hydrophilic acrylate	6.0/11.0 5.7/10.5	Refractive	Aberration-neutral	−10.0 to 1.0 (1.0) & 0 to +36.0 (0.5) +15.0 to +30.0 (0.5)	Yes (Lentis and Femtis Comfort Toric)
ACUNEX Vario	Teleon Surgical BV	Hydrophobic acrylate	6.0/12.5	Refractive	Aberration-neutral	+10.0 to +30.0 (0.5)	Yes (Acunex Vario Toric)
Asqelio EDoF	AST Products Inc.	Hydrophobic acrylate	6.0/13.0	Refractive	−0.27 μm	+5.0 to +34.0 (0.5)	Yes (Asqelio EDoF Toric)
Precizon Presbyopic	Ophtec BV	Hybrid hydrophobic & hydrophilic acrylate	6.0/12.5	Refractive	Aberration-neutral	+1.0 to +35.0 (0.5)	Yes (Precizon Presbyopic Toric)
TECNIS PureSee ZEN00V	Johnson & Johnson Vision	Hydrophobic acrylate	6.0/13.0	Refractive	−0.27 μm	+5.0 to +34.0 (0.5)	Yes (Tecnis PureSee Toric II)
Enova Advanced	VSY Biotechnology	Hydrophobic acrylate	6.0/13.0	Refractive	Aberration-neutral	+10.0 to +30.0 (0.5)	Yes (Enova Advanced Toric)
TECNIS Symfony	Johnson & Johnson Vision	Hydrophobic acrylate	6.0/13.0	Refractive–diffractive	−0.27 μm	+5.0 to +34.0 (0.5)	Yes (Tecnis Symfony Toric)
AT LARA	Carl Zeiss	Hydrophilic acrylate	6.0/11.0	Refractive–diffractive	Aberration-neutral	−10.0 to +32.0 (0.5)	Yes (AT LARA Toric)
Eden	Swiss Advanced Vision	Hydrophilic acrylate	6.0/10.8 6.0/12.4	Refractive–diffractive	Not available	+5.0 to +30.0 (0.5)	Yes (Eden MT)
IC-8 Apthera	Bausch + Lomb	Hydrophobic acrylate	6.0/12.5	Refractive–diffractive (pinhole)	Aberration-neutral	+10.0 to +30.0 (0.5)	No
Active	Hanita	Hydrophilic acrylate	6.0/11.0	Refractive–diffractive	Not available	0 to +30.0 (0.5) +30.0 to +35.0 (1.0)	No

**Table 4:** Summary of select studies on extended depth of focus intraocular lenses regarding their visual acuity findings.

Publication (Author, Year)	IOL Model	No. of Eyes	NVA (logMAR)	IVA (logMAR)	DVA (logMAR)
Auffarth, 2020 [30]	MiniWell	136	0.18 ± 0.14	0.06 ± 0.12	−0.05 ± 0.11
Campos, 2024 [31]	LuxSmart	40	0.11 ± 0.06	−0.05 ± 0.03	0.08 ± 0.04
Giannuzzi, 2024 [32]	AcrySof IQ Vivity	36	0.16 ± 0.06 (U)	0.02 ± 0.05 (U)	−0.04 ± 0.07
Pedrotti, 2023 [33]	Lucidis	50	0.07 ± 0.11	0.11 ± 0.11	−0.04 ± 0.08
Ferrando Gil, 2024 [34]	ELON	20	0.37 ± 0.05	0.27 ± 0.05	0.05 ± 0.06
Song, 2020 [35]	LENTIS comfort	47	0.36 ± 0.15	0.14 ± 0.11	0.0 ± 0.05
Rua Amaro, 2024 [36]	ACUNEX Vario	40	0.25 ± 0.08	0.03 ± 0.09	−0.09 ± 0.06
Tañá-Sanz, 2025 [37]	Asqelio EDof IOL	44	0.37 ± 0.12	0.10 ± 0.11	0.01 ± 0.06
León-Ibáñez, 2025 [38]	Precizon Presbyopic	40	0.15 ± 0.10 (B)	0.04 ± 0.05 (B)	−0.07 ± 0.06
Corbett, 2024 [39]	TECNIS PureSee	120	0.37 ± 0.10	0.13 ± 0.08	−0.06 ± 0.08
Waring, 2024 [40]	TECNIS Symfony	26	0.30 ± 0.15	0.26 ± 0.08	−0.05 ± 0.10 (U)
Reinhard, 2021 [41]	AT LARA	152	0.32 ± 0.19	0.12 ± 0.18	0.04 ± 0.16
Schojai, 2020 [42]	IC-8 Apthera	18	0.17 ± 0.11	0.03 ± 0.68	-

All studies are cited in the References. The visual acuities are all distance-corrected and monocular measurements, unless indicated by (U) in front for uncorrected or indicated by (B) for binocular measurement. IOL: intraocular lens; No.: number; NVA: near visual acuity; IVA: intermediate visual acuity; DVA: distance visual acuity.

In the refractive EDoF IOL group, MiniWell (SIFI, Catania, Italy) uses a three-zone design, each with distinct SA properties that give MiniWell its EDoF properties. The central 2 mm circular zone induces a positive SA, the next 2–3 mm ring induces a negative SA, and the last 3–6 mm has no SA properties. In a multicenter study, the MiniWell showed a mean corrected distance binocular visual acuity of 0.01 logMAR at the intermediate distance and −0.08 logMAR at the far distance [30]. Similar to the MiniWell, LuxSmart (Bausch + Lomb, Rochester, NY, USA) uses SA in such a way that its central 2 mm zone includes its fourth and sixth orders with opposite signs, next a transition zone, and then an outer monofocal zone [43]. In a study on LuxSmart IOLs compared to a control monofocal IOL, the depth of field was extended by 0.77 D at 0.2 logMAR, and the mean visual acuity at 80 cm improved from 0.36 to 0.12 logMAR while the far distance foci did not differ [44]. In another study of 40 eyes of 20 patients, follow-up at 3 months post-operation showed a monocular dominant eye CDVA of 0.08 ± 0.04, corrected intermediate VA (CIVA) of −0.05 ± 0.03, and corrected near VA (CNVA) of 0.11 ± 0.06 [31]. LuxSmart patients have reported minimal dysphotopsia, with a study finding no patients reporting the unwanted visual effect of a halo in a questionnaire [44].

In contrast to Miniwell and LuxSmart, Vivity (Alcon Inc., Fort Worth, TX, USA) utilizes a central 2.2 mm zone with a monofocal lens with increased refractive power, followed by a surface elevation—a combination that is referred to as wavefront shaping technology [28,45]. While there is not yet any literature on clinical studies for the latest Clareon Vivity model, studies on its predecessor (AcrySof IQ Vivity) report positive outcomes for its use. In a prospective single-center study involving 40 patients implanted bilaterally with AcrySof IQ Vivity, 21 (53%) reported spectacle independence at all distances [32]. In another prospective single-center study including 43 patients implanted bilaterally with this lens, binocular DCVA was reported to be lower than 0.1 logMAR at defocus between +1.0 and −1.5 D [46]. At intermediate (66 cm) and near (40 cm) foci, mean visual acuity has been reported as −0.07 to 0.10 logMAR and 0.19 to 0.29 logMAR, respectively [46,47]. In a study of Vivity, 63% of patients reported no optical phenomena, and 75% of patients reported being free of glare and halo effects [47]. Concerning all three mentioned models, optical bench studies have additionally found their performance to depend on the pupil size [28]. An optical bench study reported that Vivity, LuxSmart, and MiniWell show a decreased EDoF effect as pupil size increases, although this may be less of a concern in the general cataract population, where pupil sizes often range from 3 to 3.5 cm [48,49].

Other refractive IOLs include the Lucidis (Swiss Advanced Vision, Neuchâtel, Switzerland), which induces an additional power of +3.0 D using its 1 mm aspheric central zone [33]. The Lucidis was found to have a binocular distance-corrected VA of 0.00 ± 0.08 logMAR at near, 0.04 ± 0.09 logMAR at intermediate, and −0.07 ± 0.09 logMAR at far distances [33]. The ELON (Medicontur, Zsámbék, Hungary) and Basis Z (1stQ, Mannheim, Germany) use “wavefront linking”, meaning that they create zones of various refractive powers by changing the curvature in a 20 μm transition zone, in the central 2 mm region. In a prospective study of 10 patients implanted bilaterally with Bi-Flex POB-MA 877PEY (ELON), patients were reported to have a mean CDVA of 0.02 (±0.03), a mean CIVA of 0.29 (±0.08), and a mean CNVA of 0.40 (±0.05) logMAR [34]. The LENTIS Comfort and ACUNEX Vario (Teleon Surgical BV, Spankeren, The Netherlands) feature a three-zone design including distance, transition, and +1.5 D zones. The LENTIS Comfort is hydrophilic, while the ACUNEX Vario is hydrophobic. The LENTIS Comfort has been found to have a distance VA of 0.00 ± 0.05 logMAR and an intermediate VA of 0.14 ± 0.11 logMAR at 80 cm [35]. However, in the same study, it was found to have similar outcomes to a monofocal control IOL for near visual acuity at 40 cm [35].

Two prospective clinical studies on ACUNEX Vario have reported mean binocular uncorrected visual acuity of −0.08 to 0.00 logMAR at distance, −0.03 to 0.04 logMAR at intermediate, and 0.16 to 0.22 logMAR at near [36,50]. In one of the studies where ACUNEX Vario was compared to the AcrySof IQ Vivity IOL (discussed later in the EDoF section), their contrast sensitivity, reading speeds, and optical side effects were comparable [50]. The Asqelio EDoF IOL (AST Products Inc., Billerica, MA, USA) is a bi-aspheric lens that uses a Phase-Ring technology. In a clinical study, the average postoperative spherical equivalent was reported to be −0.31 D (±0.30 D) [37]. All patients reported a CDVA of 20/25 or better and a DCIVA of 20/32 or better, with a light distortion index similar to published studies of monofocal and diffractive EDoF IOLs [37].

Precizon Presbyopic (Ophtec BV, Groningen, The Netherlands) is a refractive IOL with a multi-segment design providing a transitional focus over the extended range of vision. In a case series of 40 eyes of 20 patients aged 47 to 81 years old, patients respectively had an uncorrected distance visual acuity (UDVA), DCIVA, uncorrected near visual acuity (UNVA), and DCNVA of −0.09 ± 0.07, 0.04 ± 0.05, 0.17 ± 0.12, and 0.15 ± 0.10 logMAR at a mean follow-up of 12.1 months [38]. In another study of 56 patients with a mean age of 65.0 years old implanted with Precizon Presbyopic IOL, 75% of patients reported no glare, 68% reported no halos, and 55% reported no starbursts at 6 months postoperatively [51].

Lastly, the newest refractive EDoF IOL is the TECNIS PureSee ZEN00V (Johnson & Johnson Vision, Irvine, CA, USA). This IOL exhibits a biconvex design, has a wavefront-modified anterior aspheric surface, and induces depth of focus extension using a continuous-power technology [39,52]. In clinical studies, the TECNIS PureSee has shown a monocular DCNVA of 0.37 ± 0.10 logMAR and DCIVA (at 66 cm) of 0.13 ± 0.08 logMAR [39]. Two optical bench studies with this IOL have shown similar results with predicted VA of ~0.15 to ~0.20 logMAR at −2.2 D, and ~0.05 to ~0.08 logMAR at −1.5 D [52,53]. Another optical bench study comparing Clareon Vivity to TECNIS PureSee found them to have comparable results at intermediate and far distances, with predicted visual acuities of −0.04 vs. −0.05 logMAR at 0 D and 0.05 vs. 0.07 logMAR at −1.50 D of defocus, respectively [54]. The study also found nearly identical results in introducing the unwanted side effect of visualizing a halo pattern when assessed by the point spread function intensity profile [54]. Clinical studies report low levels of dysphotopsia, with 87% to 93% of patients not experiencing halos, 95% to 100% not reporting glares, and 95% to 100% not reporting starbursts [39,52,55]. Recent additions to the refractive EDoF IOL category also include the Enova Advanced (VSY Biotechnology, Leinfelden-Echterdingen, Germany) and LuxLife (Bausch + Lomb, Bridgewater, NJ, USA). However, no peer-reviewed clinical results have been published to date, with a multicenter trial of the latter model having only recently been completed [56].

In addition to the refractive EDoF IOLs, diffractive models are also available. TECNIS Symfony (Johnson & Johnson Vision, Irvine, CA, USA) is a diffractive IOL with nine zones that uses first- and second-order diffraction for distance and intermediate focus, respectively, and corrects chromatic and spherical aberrations [57,58]. In a clinical study of patients implanted with TECNIS Symfony OptiBlue (DXR00V IOL), the mean monocular DCIVA at 66 cm was 0.26 ± 0.08 logMAR, while the mean DCNVA was 0.30 ± 0.15 logMAR at 33 cm [40]. In another clinical study, eyes implanted with DXR00V TECNIS Symfony OptiBlue IOL showed better distance-corrected reading performance at intermediate distances but lower performance at near distances compared to the DFR00V TECNIS Synergy IOL [59]. Lastly, a retrospective clinical study found that for patients with a mild myopic target, Symfony EDoF IOL can enhance near vision, reported to be an uncorrected VA of 0.18 logMAR with minimal impacts on intermediate and distance vision, respectively reported to be 0.07 and 0.03 logMAR [60]. Similar to the technology used by TECNIS Symfony, AT LARA (Carl Zeiss, Jena, Germany) contains 13 zones and offers a 1.90 D addition for the intermediate focus [58]. In a multicenter, randomized study comparing AT LARA and TECNIS Symfony, the two lenses were found to be similar in binocular depth of field [41], although compared to the control group, both lenses performed superiorly. Binocular DCIVA at 67 cm was 0.05 ± 0.15 with AT LARA and 0.08 ± 0.14 logMAR with TECNIS Symfony, compared to 0.29 ± 0.15 logMAR in the control group [41]. However, the performance of these two diffractive IOLs is impacted by the color of the light [58,61,62]. In red light, the far focus is enhanced while the intermediate focus deteriorates, and in blue light, the lenses act as monofocal lenses [58,62]. Therefore, while such diffractive EDoF IOLs have the advantage of correcting chromatic aberration, this comes at the expense of visual perception under spectral conditions that deviate significantly from white-light illumination.

Lastly, the Eden EDoF IOL (Swiss Advanced Vision, Neuchâtel, Switzerland) is a diffractive IOL with a 1 mm central aspheric area, with a diffractive pattern that directs the light to +3.0 D near and distance focus. The Active IOL (Hanita, Israel) employs a diffractive pattern with an add power of +2.25 D, with no peer-reviewed publication available on its technology and functional outcomes. The IC-8 Apthera IOL (Bausch + Lomb, Rochester, NY, USA) is another diffractive EDoF design. Instead of diffractive steps, this IOL uses a small aperture approach to expand the depth of field: the monofocal lens contains a 5 μm thin opaque disk with a 1.36 mm opening [63]. In a clinical study comparing the TECNIS Symfony and the IC-8 Apthera using the micro-monovision approach, the study revealed a significantly superior UDVA but similar intermediate VA for the IC-8 Apthera compared to the Symfony group [42]. Regarding their unwanted visual side effects, this study found that a larger proportion of Symfony patients reported halos (63% vs. 31%), blurred vision (44% vs. 25%), and diurnal fluctuations in visual acuity (44% vs. 19%) compared to the IC-8 group [42].

## 5. Trifocal IOLs

Trifocal IOLs provide an enhanced near visual acuity compared to EDoF IOLs by splitting the light into three primary foci, leading to a higher chance of spectacle independence [64]. These IOLs are often fully diffractive, with the degree of apodization directly correlating with the degree of pupil independence [65,66]. A summary of the characteristics of the trifocal IOLs is included in Table 5. A summary of the select studies on these lenses, when available, is included in Table 6.

**Table 5:** Overview of trifocal intraocular lenses’ characteristics.

Model	Manufacturer	Material	Optics Diameter/ Overall Diameter (mm)	Optical Design	Spherical Aberrations	Available Strengths (Steps) (D)	Availability of Toric Versions
RayOne Galaxy	Rayner	Hydrophilic acrylate	6.0/12.5	Refractive	−0.17 μm	+5.0 to +30.0 (0.5)	Yes (RayOne Galaxy Toric)
LuxLife	Bausch + Lomb	Hydrophilic acrylate	6.0/11.0	Refractive	Not available	+5.0 to +10.0 (1.0) +10.5 to +30.0 (0.5) +31.0 to +32.0 (1.0)	Yes (LuxLife Toric)
FineVision POD F/ POD F GF	BVI Medical	Hydrophilic acrylate/ hydrophobic acrylate	6.0/11.4	Refractive–diffractive	−0.11 μm	+6.0 to +35.0 (0.5)/+10.0 to +35.0 (0.5)	Yes (FineVision POD FT/POD FT 49P)
AT LISA tri	Carl Zeiss	Hydrophilic acrylate	6.0/11.0	Refractive–diffractive	−0.18 μm	0 to +32.0 (0.5)	Yes (AT Lisa tri Toric)
AT ELANA	Carl Zeiss	Hydrophobic acrylate	6.0/13.0	Refractive–diffractive	Aberration-neutral	0 to +34.0 (0.5)	No
PanOptix (Clareon)	Alcon Inc.	Hydrophobic acrylate	6.0/13.0	Refractive–diffractive	−0.10 μm	+6.0 to +30.0 (0.5) +31.0 to +34.0 (1.0)	Yes (PanOptix Toric)
RayOne Trifocal	Rayner	Hydrophilic acrylate	6.0/12.5	Refractive–diffractive	Aberration-neutral	0 to +30.0 (0.5)	Yes (RayOne Trifocal Toric)
Acriva Trinova	VSY Biotechnology	Hydrophilic acrylate	6.0/13.0	Refractive–diffractive	−0.10 μm	0 to +32.0 (0.5)	Yes (Acriva Trinova Toric)
Triva aAY	HumanOptics	Hydrophilic acrylate	6.0/12.5	Refractive–diffractive	Aberration-neutral	+10.0 to +30.0 (0.5)	Yes (TrivaT-aAY)
Triva aXAY	HumanOptics	Hydrophilic acrylate	7.0/11.0	Refractive–diffractive	Aberration-neutral	+10.0 to +30.0 (0.5)	Yes (TrivaT-aXAY)
Intensity	Hatina Lenses	Hydrophilic acrylate/ hydrophobic acrylate (HP)	6.0/11.0 (BN) 6.0/13.0	Refractive–diffractive	−0.13 μm	+10.0 to +30.0 (0.5) +17.5 to +27 (0.5) (HP)	Yes (Intensity Toric)
Vivinex Gemetric/ Gemetric Plus	Hoya Surgical Optics	Hydrophobic acrylate	6.0/13.0	Refractive–diffractive	−0.16 μm	+10.0 to +30.0 (0.5)	Yes (Vivinex Gemetric Toric/Gemetric Plus Toric)
Liberty 677	Medicontur Medical Engineering Ltd.	Hydrophilic acrylate	6.0/13.0	Refractive–diffractive	Aberration-neutral	+8.0 to +35.0 (0.5)	Yes (Liberty Toric 677)
TECNIS Synergy	Johnson & Johnson Vision	Hydrophobic acrylate	6.0/13.0	Refractive–diffractive	−0.27 μm	+5.0 to +34.0 (0.5)	Yes (TECNIS Synergy Toric II)
TECNIS Odyssey	Johnson & Johnson Vision	Hydrophobic acrylate	6.0/13.0	Refractive–diffractive	−0.27 μm	+5.0 to +34.0 (0.5)	Yes (TECNIS Odyssey Toric II)
enVista Envy	Bausch + Lomb	Hydrophobic acrylate	6.0/12.5	Refractive–diffractive	−0.15 μm	+6.0 to +10.0 (1) +10.0 to +34.0 (0.5)	Yes (enVista Envy Toric)
Asqelio Trifocal	AST Products Inc.	Hydrophobic acrylate	6.0/13.0	Refractive–diffractive	−0.27 μm	+5.0 to +34.0 (0.5)	Yes (Asqelio Trifocal Toric)
Optiflex Trio	Biotech Healthcare	Hydrophobic acrylate	6.0/13.0	Refractive–diffractive	−0.2 μm	+7.0 to +30.0 (0.5)	Yes (OptiFlex Trio 3FLA6T)

**Table 6:** Summary of select studies on trifocal intraocular lenses regarding their visual acuity findings.

Publication (Author, Year)	IOL Model	No. of Eyes	NVA (logMAR)	IVA (logMAR)	DVA (logMAR)
Ang, 2023 [67]	FineVision POD F	48	0.08 ± 0.10 (B)	0.04 ± 0.08 (B)	0.03 ± 0.05 (B)
Ang, 2023 [67]	FineVision POD F GF	44	0.06 ± 0.09 (B)	0.04 ± 0.08 (B)	−0.01 ± 0.05 (B)
Chen, 2024 [68]	AT LISA tri	164	0.05 ± 0.09	0.05 ± 0.09	−0.03 ± 0.07
Mendicute, 2024 [69]	Clareon PanOptix	76	0.15 ± 0.11	0.11 ± 0.14	−0.02 ± 0.08
Llovet-Rausell, 2024 [70]	RayOne Trifocal	8432	0.10 ± 0.08 (BU)	0.13 ± 0.11 (BU)	0.04 ± 0.08 (BU)
Kılıç, 2025 [71]	Acriva Trinova Pro C	202	0.14 ± 0.12	0.14 ± 0.14	0.01 ± 0.03
Pastor-Pascual, 2023 [72]	Triva-aXAY	46	0.07 ± 0.07	0.10 ± 0.06	0.03 ± 0.05
Bianchi, 2025 [73]	Intensity L	120	0.07 ± 0.07	0.03 ± 0.06	−0.05 ± 0.06
Kaymak, 2024 [74]	Vivinex Gemetric	72	≈0.12 ± 0.13	≈0.11 ± 0.12	≈−0.04 ± 0.12
Kaymak, 2024 [74]	Vivinex Gemetric Plus	104	≈0.07 ± 0.11	≈0.12 ± 0.10	≈0.0 ± 0.09
Villarrubia Cuadrado, 2022 [75]	Liberty 677	26	0.13 ± 0.10	0.22 ± 0.16	0.01 ± 0.15
Baur, 2023 [76]	TECNIS Synergy	56	−0.01 ± 0.06	−0.03 ± 0.09	−0.09 ± 0.05
Shultz, 2025 [77]	enVista Envy	332	0.15 ± 0.01	0.12 ± 0.01	0.03 ± 0.01
Palomino-Bautista, 2022 [78]	Asqelio Trifocal	50	0.04 ± 0.08	0.03 ± 0.08	−0.05 ± 0.06
Brar, 2021 [79]	Optiflex Trio	54	−0.01 ± 0.03 (B)	0.06 ± 0.05 (B)	−0.06 ± 0.04 (B)

All studies are cited in the References. The visual acuities are all distance-corrected, unless indicated by (U) in front for uncorrected. (B) indicates binocular measurement. IOL: intraocular lens; No.: number; NVA: near visual acuity; IVA: intermediate visual acuity; DVA: distance visual acuity.

FineVision IOL, initially produced by PhysIOL and now BVI Medical (Waltham, MA, USA), was one of the first such trifocal IOLs. Its two designs, POD F and POD F GF, use a diffractive pattern with a pupil-dependent asymmetric light energy distribution that can lead to increased distance visual acuity in low-light conditions [80]. Clinical studies have shown both to have comparable results, with CDVA of 0.03 ± 0.05 logMAR for POD F and −0.01 ± 0.05 logMAR for POD F GF, DCIVA at 70 cm of 0.04 ± 0.08 logMAR for POD F and 0.04 ± 0.08 logMAR for POD F GF, and lastly DCNVA at 35 cm of 0.08 ± 0.10 logMAR for POD F and 0.06 ± 0.09 logMAR for POD F GF [67].

Another trifocal model is the AT LISA tri (Carl Zeiss, Jena, Germany), which demonstrates pupil independence up to a pupil size of 4.34 mm, with pupil sizes above this value leading to improved distance visual acuity at the expense of intermediate visual acuity [68]. In a study of AT LISA tri 839 MP in 164 eyes of presbyopic patients, 100%, 90.2%, and 89.0% of patients achieved binocular UDVA, UNVA, and uncorrected intermediate VA (UIVA) of at least logMAR 0.1 at 6 months post-implantation [68]. Halo was reported in 66.2% of the patients, followed by glare in 18.2% and starburst in 7.8% of patients [68]. Similar to AT LISA tri, AT ELANA (Carl Zeiss, Jena, Germany) uses the same diffractive principles but is hydrophobic and has a different haptic configuration [81,82]. In an optical bench study comparing the AT LISA tri and AT ELANA using the MTF, simulated visual acuity, and light distribution around a point light source, the two models were found to have similar optical qualities and photic phenomena [82]. Both models demonstrated a simulated visual acuity of 0.0 logMAR for far vision, 0.10 logMAR at 80 cm, and 0.05 logMAR at 40 cm [82].

The PanOptix (Alcon Inc., Fort Worth, TX, USA) is a non-apodized diffractive trifocal lens, with its diffractive zones expanding across the 4.5 mm lens surface [65]. Compared to other trifocal lenses, PanOptix was found to have close intermediate and near foci that merge into a single extended focus [64,66]. In a study comparing AcrySof IQ PanOptix to AT LISA tri 839 MP, PanOptix was found to have a better UIVA of 0.23 vs. 0.31 logMAR and had higher contrast sensitivity under both photopic and mesopic conditions with and without glare [83]. In another study comparing the AcrySof IQ PanOptix to a diffractive bifocal (ZMB00, Johnson & Johnson Vision, Irvine, CA, USA), UIVA and contrast sensitivity were significantly better in the trifocal group, whereas the UNVA and higher-order aberrations were better in the bifocal group [84]. Lastly, in two prospective longitudinal studies with Clareon PanOptix, at 1 month post-operation, mean monocular CDVA, CIVA, and CNVA were 0.00 ± 0.09, 0.02 ± 0.17, and 0.12 ± 0.12 logMAR, respectively, while the mean postoperative binocular CDVA, DCIVA (at 60 cm), DCNVA (at 40 cm), and DCNVA (at 33 cm) were −0.04 ± 0.08 logMAR, −0.08 ± 0.10 logMAR, 0.01 ± 0.10 logMAR, and 0.04 ± 0.11 logMAR, respectively [69,85]. In one study, 100% of patients reported no hazy vision, while in the other one, 9% of patients were bothered at least “quite a bit” by halos, followed by 12% and 0% of patients bothered at least “quite a bit” by starburst and glare, respectively [69,85].

The RayOne trifocal (Rayner, Worthing, West Sussex, UK) has a 4.5 mm diffractive zone with two profiles. At 3 months post-operation, patients implanted bilaterally with RayOne trifocal had a binocular UDVA of 0.04 ± 0.08 logMAR, monocular CDVA of 0.05 ± 0.07 logMAR, binocular UNVA of 0.10 ± 0.08 logMAR, and binocular UIVA of 0.13 ± 0.11 logMAR [70]. The RayOne Galaxy (Rayner, Worthing, West Sussex, UK) is a biconvex aberration-correcting IOL, made from a single-piece Rayacryl hydrophilic acrylic material. It has a non-diffractive spiral design, with add powers of +1.5 D and +3.0 D. Through its optical design, the lens yields binocular defocus VA of at least 0.2 logMAR across a range of approximately 4.0 D [86]. Looking at 25 patients reported in a clinical investigation by Rayner, CDVA at 0 D was reported at approximately −0.05 logMAR, a DCIVA of 0 logMAR was reported at −1.5 D, and lastly DCNVA at −2.8 D was reported to reach 0.2 logMAR [86]. The manufacturer also reported patients implanted with Galaxy to have minimized dysphotopsia compared to diffractive trifocals using a halo and gale simulated at 1 month post-operation [86].

Acriva Trinova (VSY Biotechnology, Leinfelden-Echterdingen, Germany) has a classic light distribution with a 3 mm pupil, but with larger pupil sizes, intermediate visual acuity improves while the near visual acuity declines. In studies, Acriva Trinova has been found to have mixed results regarding visual acuity compared to PanOptix. While Acriva Trinova has been shown to have worse visual acuity than PanOptix [65,71], it has also been found to have a significantly better visual acuity at 80 cm despite similar performance in other foci or contrast sensitivity [87]. A prospective multicenter study of 101 patients with bilateral implantation of the Trinova Pro C IOL (VSY Biotechnology, Leinfelden-Echterdingen, Germany) showed that at 6 months post operation, mean monocular logMAR CDVA, DCIVA, and DCNVA were 0.01 ± 0.03, 0.14 ± 0.14, and 0.14 ± 0.12 logMAR, respectively [88].

The TRIVA-aAY and TRIVA-aXAY (HumanOptics, Erlangen, Germany), with 6 mm and 7 mm optic diameter, respectively, display pupil dependence. In a clinical study, TRIVA-aXAY was found to have a binocular CDVA, DCIVA (60 cm), and DCNVA (40 cm) of 0.00 ± 0.05, 0.06 ± 0.06, and 0.02 ± 0.05 logMAR, respectively [72]. The Intensity (Hanita Lenses, Shlomi, Israel) is a pentafocal lens that has five symmetrically distributed orders of diffraction, to allow a smooth transition across multiple distances [89]. Alió et al. in an observational prospective longitudinal study of 64 eyes implanted bilaterally with Intensity found a maximum vision at distance of 0.02 ± 0.07 logMAR at 0 D, with decline through intermediate (0.11 ± 0.08 logMAR at −1.5 D) and near vision (0.12 ± 0.12 logMAR at −2.5 D) [90]. A study by Bianchi, looking at 120 eyes of 60 patients with a mean age of 68.32 years with bilateral implantation of Intensity, found a UIVA at 120 cm of 0.06 ± 0.11 logMAR, a UIVA at 80 cm of 0.07± 0.07 logMAR, a UIVA at 66 cm of 0.05 ± 0.07 logMAR, and a UNVA at 40 cm of 0.09 ± 0.08 logMAR at 24 months follow-up [73].

The Vivinex Gemetric (Hoya Surgical Optics, Tokyo, Japan) and Vivinex Gemetric Plus (Hoya Surgical Optics, Tokyo, Japan) have a central 3.2 diffraction pattern, with the rest being monofocal. In a prospective randomized multicenter trial comparing 36 and 52 patients implanted with Gemetric or Gemetric Plus lenses, respectively, the Gemetric Plus group showed a better UNVA at about 0.05 logMAR compared to 0.08 logMAR of the Gemetric group [74]. However, the Gemetric group performed superiorly to the Gemetric Plus group at intermediate (UIVA 0.03 vs. 0.07 logMAR) and far distances (UDVA −0.04 vs. 0.01 logMAR) distances [74]. The Liberty 677 (Medicontur Medical Engineering Ltd., Zsámbék, Hungary) contains a 3 mm diffraction zone that distributes light into the distance and near focus. The intermediate focus is achieved through constructive interference between both orders. In a study with the Liberty 677, uncorrected distance, intermediate, and near visual acuity were −0.03 ± 0.13, 0.21 ± 0.16, and 0.16 ± 0.09 logMAR, respectively [75]. In another prospective single-center study of 29 patients, 100%, 92%, and 80% of patients achieved a postoperative binocular UDVA, UIVA, and UNVA of 20/25 or better, respectively, at 3 months postoperatively [9]. Regarding the side effects, 83.3%, 83.4%, and 83.3% of patients respectively reported no bothersome to moderately bothersome halo, starburst, and glare [9].

The TECNIS Synergy (Johnson & Johnson Vision, Irvine, CA, USA) reduces chromatic aberration at each focal point. In a clinical study, the peaks of the defocus curve were −1.5 D for the intermediate and −3.0 D for the near focus [91], and the binocular visual acuities were found to be −0.13 ± 0.06 logMAR at distance, −0.09 ± 0.06 logMAR at 80 cm, and −0.05 ± 0.06 logMAR at 40 cm [76]. The preferred reading distance for patients with Synergy implantation was found to be 36.9 ± 3.0 cm [76]. The newest version of the TECNIS Synergy is the TECNIS Odyssey (Johnson & Johnson Vision, Irvine, CA, USA). Based on reports by Johnson & Johnson Vision, 93% of the patients implanted with Odyssey reported no or mild halos, glare, or starbursts at the one-month follow-up. Its simulated visual acuity was close to 20/18 at 0 D, 20/25 at 1.5 D, and 20/22 at −2.5 D [92].

The enVista Envy IOL (Bausch + Lomb, Bridgewater, NJ, USA) is a refractive–diffractive trifocal intraocular lens with intermediate and near add powers of 1.6 D and 3.1 D, respectively [93]. The lens features an apodized diffractive profile designed to improve distance vision and reduce the impact of photic phenomena under low-light conditions. In a randomized US clinical trial by Shultz et al., 332 patients received bilateral Envy implants, while 169 received a monofocal lens as a control group [93]. The Envy group achieved a monocular DCVA of 0.032 ± 0.006 logMAR, compared to −0.008 ± 0.008 logMAR for controls, confirming non-inferiority for distance vision. Notably, there was a clear improvement at intermediate (66 cm) and near (40 cm) distances, with Envy showing 0.124 ± 0.009 vs. 0.352 ± 0.012 logMAR, and 0.146 ± 0.010 vs. 0.541 ± 0.013 logMAR, respectively, compared to monofocal, with further improvements seen binocularly. Comparable results were reported in a Canadian multicenter clinical trial involving 165 patients, where 67% received the Envy and 33% a monofocal IOL [77]. Monocular DCVA was 0.02 ± 0.12 vs. −0.04 ± 0.09 logMAR, DCIVA was 0.15 ± 0.12 vs. 0.34 ± 0.13 logMAR, and DCNVA was 0.18 ± 0.13 vs. 0.53 ± 0.14 logMAR for the Envy and monofocal IOL, respectively. Both trials demonstrated an extended range of vision with a flat defocus curve and high postoperative satisfaction among patients implanted with the Envy model.

Asqelio Trifocal (AST Products Inc., Billerica, MA, USA) is a bi-aspheric diffractive–refractive model, which provides 2.2 D and 3.3 D add powers. The lens contains 15 diffractive steps within the central 4.5 mm zone with an overall optical diameter of 6 mm. In a study by Palomino-Bautista et al., which involved 25 patients (50 eyes), a monocular DCVA of −0.05 ± 0.06 logMAR was reported, with good VA preserved at intermediate (67 cm, 0.03 ± 0.08 logMAR) and near distances (40 cm, 0.04 ± 0.08 logMAR) [94]. It was supported by the results from other studies, where Tañá-Sanz et al. reported far, 67 cm, and 40 cm monocular distance-corrected VA of 0.01 ± 0.06, 0.10 ± 0.11, 0.37 ± 0.12 logMAR and 0.03 ± 0.08, 0.09 ± 0.11, 0.09 ± 0.11 logMAR, in 2024 and 2025, respectively [37,78]. Despite the perception of photic phenomena, 90.9% of patients post-surgery reported being satisfied with their vision [37], with at least an 8/10 subjective score reported by 72% of subjects of Palomino-Bautista et al. [94].

The Optiflex Trio (Biotech Healthcare, Ahmedabad, Gujarat, India) is a diffractive–refractive trifocal IOL with near and intermediate additions of +3.5 D and +1.85 D, respectively, and a close energy split between intermediate and near foci. The model features diffractive rings spanning the central 4 mm portion of the lens, with the peripheral part being purely refractive. At 12 months postoperatively, Brar et al. reported binocular DCVA of −0.06 ± 0.04 logMAR at 4 m, 0.02 ± 0.04 logMAR at 80 cm, 0.06 ± 0.05 logMAR at 60 cm, and −0.01 ± 0.03 logMAR at 40 cm [95]. In that study, patients were evaluated at multiple follow-up times up to 12 months, showing stable visual outcomes with no significant differences across time points. The satisfaction rate was high, i.e., 97.07 ± 2.23%, but more studies are needed, as only 27 patients were enrolled in this evaluation [95].

## 6. Individualized Approaches

Individualized IOL selection should consider every patient’s specific needs—ranging from their visual expectations to their concurrent or previous ocular history. Patients’ visual needs may vary depending on their desired lifestyles and their tolerance to side effects such as glares, halos, or starbursts. For example, patients who routinely rely on their computers may benefit from IOLs with good visual acuity outcomes reported at the intermediate distance [79]. Or, for example, if a patient routinely drives at night, they may prefer to choose IOLs that offer lower rates of photic phenomena [96,97]. Patients’ ocular history additionally impacts their surgical outcomes. Patients with larger pupils may experience higher dysphotopsia rates, especially using multifocal IOLs [98]. Squint history, poor stereopsis or amblyopia reduce the chances of a satisfactory outcome with presbyopia-correcting IOLs, particularly in complementary IOL systems designed for binocular implantation [99]. However, a mix-and-match approach may be considered in such patients.

Dry eye disease is another common issue that impacts IOL implantation. Such patients’ unstable tear film may lead to errors in corneal astigmatism power and axis preoperative assessments [97]. These patients may also be dissatisfied after multifocal diffractive IOL implantation, as it leads to fluctuating vision quality [100]. Therefore, for patients with dry eye, non-diffractive IOL models are preferable. Other ocular comorbidities, which may progress with time, must also be considered. Unstable capsular bag—for example in pseudoexfoliation syndrome or high myopia—may predispose to IOL decentration. This is particularly relevant for some IOL models that rely on precise centration, such as diffractive multifocal IOLs. Furthermore, patients with mild epiretinal membranes or early stages of age-related macular degeneration should be counseled about possible loss of contrast sensitivity and limited visual prognosis [99].

Previous corneal refractive surgery introduces additional complexity in IOL selection, as procedures such as LASIK or PRK can alter corneal curvature and subsequently affect SA [101]. Myopic LASIK or PRK typically increases positive spherical aberration, whereas hyperopic LASIK tends to reduce it [102]. Therefore, patients with a history of myopic LASIK may benefit from IOLs with negative SA, while those who have undergone hyperopic LASIK are generally better suited for aberration-neutral IOLs or even IOLs with positive SA, e.g., spherical models. The computational simulation of AcrySof Vivity performance in post-myopic LASIK surgery eyes showed larger depth of focus for small pupils and smaller halo for large pupils [102]. Good visual outcomes and patient satisfaction were also reported in a clinical study of Vivity implantation in post-myopic refractive surgery patients [103]. Overall, in patients who have undergone corneal refractive procedures, presbyopia-correcting IOLs may be considered for eyes with a well-centered ablation and lower amounts of higher-order aberrations [97].

The recommendation to define specific cut-off pupil size values for guiding IOL selection must be based on clinical evidence. Although pupil dependency is largely determined by the optical design of the IOL, the interaction within the eye is complex and cannot be generalized to every patient. Still, when interpreting optical bench results in relation to their clinical impact, it is important to recognize that the pupil sizes reported in optical bench studies usually refer to the pupil size at the IOL plane (i.e., an aperture stop), whereas clinically, a magnified image of this aperture is measured. This leads to a systematic difference, such that, for example, a 3.0 mm pupil at the IOL plane corresponds to approximately 3.5 mm clinically, and a 4.5 mm pupil at the IOL plane corresponds to approximately 5.2 mm clinically. In clinical evaluation of the pupil size impact on the Eyhance IOL, it was found that binocular distance-corrected intermediate visual acuity and pupil size were significantly correlated (with a Spearman Rho ratio of 0.383) [104]. In another study on Eyhance, with a 2 mm entrance pupil, the curve showed a single broad peak centered at −0.6 D [105]. With pupil size at 3.5 mm, the peak centered at 0 D. And for larger pupils, the far vision peak remained stable [105]. In trifocal IOLs, for example, a clinical study on AcrySof IQ PanOptix found visual performance to be mainly independent of pupil sizes [106]. However, smaller pupils were found to yield better visual acuity across the defocus curve, and a trend of patients being more satisfied with newspaper reading was observed [106]. Such clinical studies, and their interpretation supported by the optical bench data, provide further insight into the IOL performance and help guide IOL selection.

In addition to all patient-specific factors relevant to IOL selection, patients’ individualized need for an IOL can be customized through a variety of methods to reach the desired optical quality, including using sulcus-fixated or capsulotomy-fixated IOLs, and binocular or mix-and-match models. In sulcus-fixated, “reversible”, trifocal IOLs, patients can have a monofocal IOL implanted in the capsular bag and a trifocal IOL implanted in the ciliary sulcus [107]. By doing so, the trifocal may be more easily removed or exchanged [108]. For this reason, this strategy may be beneficial in situations where predicting patient satisfaction may be more challenging, such as in patients with a history of strabismus surgery, mild amblyopia, unilateral IOL implantation, or prior monofocal IOL who now wish to be spectacle-free [107]. Various methods for reversible trifocal IOLs currently available are the DUET procedure using the Rayner Sulcoflex Trifocal 703F IOL (Rayner, Worthing, West Sussex, UK), and the Liberty-2-Two-lens system (1stQ GmbH, Mannheim, Germany) [109,110].

Another method is capsulotomy-fixated IOLs, which is beneficial as the precise fixation of the lens can help withstand the change in the capsular bag size or shape that can lead to tilt, decentration, or rotation of IOLs [111]. One such IOL is the Femtis IOL (Teleon Surgical BV, Spankeren, The Netherlands) [111]. This lens has four additional haptics that are positioned anterior to the capsulotomy, in addition to the two-plate haptics that are placed in the capsular bag. A prospective clinical study on the Femtis lens has found a mean rotation of 1.50 degrees, a horizontal tilt of 0.70 degrees, a vertical tilt of 1.15 degrees, and a decentration of 0.14 mm one year after the surgery [111]. While capsulotomy-fixated Femtis offers such advantages, its use may be limited by a lack of femtosecond lasers that may not always be available for its placement.

In addition to fixing the IOLs, patients may benefit from mix-and-match concepts, in which two different IOLs are implanted in one patient [80]. This approach can be helpful in patients with complex cases, such as those with corneal irregularities or histories of unilateral IOL implantation, who may not achieve the desired visual outcomes with the planned IOL. The mix-and-match model can also help increase the depth of the field or reduce photic phenomena [80]. The results of the mix-and-match models in the literature are supportive of its use [112,113,114,115]. For example, in a study of implantation of Hoya Vivinex XC1-SP (Hoya Surgical Optics, Tokyo, Japan) in the dominant eye and a rotationally asymmetric refractive bifocal EDoF IOL with an additional power of +1.5 D (LENTIS Comfort-LS-313 MF15, Teleon Surgical BV, Spankeren, The Netherlands) in the non-dominant eye, the mean postoperative binocular UDVA, UIVA, and UNVA were 0.05 ± 0.09, −0.08 ± 0.11, and 0.06 ± 0.07 logMAR, respectively [112].

The binocular approach is derived from the mix-and-match model, where one bifocal or EDoF is used for distance and intermediate vision while another IOL is used to optimize distance and near vision. Two models have been established for the binocular approach specifically: the ARTIS Symbiose system (Cristalens, Lannion, France), where the ARTIS Symbiose MID uses its diffractive EDoF profile to create an additional intermediate focus, while the PLUS model provides for the near vision. A study of the ARTIS Symbiose system implanted in 27 patients found a mean binocular defocus curve of 0.0 logMAR or better in the +0.50 to −2.50 D range [116]. In another study of 44 patients evaluated at 12 months, binocular UDVA was −0.07 ± 0.06 logMAR, UIVA was 0.03 ± 0.10 logMAR, and UNVA was 0.07 ± 0.08 logMAR [117]. The non-diffractive model is the refractive WELL-Fusion concept (SIPI, Catania, Italy), which consists of the Mini Well EDoF IOL and the Mini Well Proxa. Both approaches have been reported to provide good visual acuity over a range of distances [116,118]. In a prospective two-center study by Mastropasqua et al. [119], including 30 patients, the binocular UDVA at 90 days was 0.03 ± 0.11 logMAR, UIVA was 0.05 ± 0.10 logMAR, and UNVA was 0.06 ± 0.08 logMAR [118].

## 7. Conclusions

With the introduction of all the new IOLs for presbyopia management, choosing a lens that meets individualized patient needs has been more challenging for both physicians and patients. This review aims to provide a comprehensive overview of the forty-seven available lenses, along with their laboratory and clinical studies. Monofocal plus IOLs demonstrate an improvement in depth of field compared to monofocal lenses, while the extended depth of focus IOLs show improved intermediate visual acuity. Trifocal IOLs offer the best visual acuity at all three foci: near, intermediate, and distance. Based on patients’ needs, strategies such as sulcus fixation or capsulotomy fixation can be used to optimize the placement and exchange of the IOLs. Mix-and-match and binocular IOL systems can additionally help patients reach spectacle independence.

## Data Availability

No new data were created or analyzed in this study.

## Abbreviations

The following abbreviations are used in this manuscript:

IOL	Intraocular lens
EDoF	Extended depth of focus
SA	Spherical aberration
CDVA	Corrected distance visual acuity
DCIVA	Distance-corrected intermediate visual acuity
MTF	Modulation transfer function
DCNVA	Distance-corrected near visual acuity
CIVA	Corrected intermediate visual acuity
CNVA	Corrected near visual acuity
UDVA	Uncorrected distance visual acuity
UNVA	Uncorrected near visual acuity
UIVA	Uncorrected intermediate visual acuity
